# Safety, activity, and pharmacokinetics of camrelizumab in advanced Asian melanoma patients: a phase I study

**DOI:** 10.1186/s12885-022-09663-5

**Published:** 2022-05-20

**Authors:** Li Zhou, Xiaowen Wu, Zhihong Chi, Lu Si, Xinan Sheng, Yan Kong, Lili Mao, Bin Lian, Bixia Tang, Xieqiao Yan, Xuan Wang, Xue Bai, Siming Li, Xiaoting Wei, Juan Li, Qing Yang, Jun Guo, Chuanliang Cui

**Affiliations:** 1 Key Laboratory of Carcinogenesis and Translational Research (Ministry of Education/Beijing), Department of Renal Cancer and Melanoma, Peking University Cancer Hospital & Institute, 52 Fucheng Road, Haidian District, Beijing, 100142 China; 2 Clinical Research & Development, Jiangsu Hengrui Pharmaceuticals Co, Ltd, Shanghai, 201203 China

**Keywords:** Camrelizumab, Advanced melanoma, Immunotherapy, Anti-PD-1 antibody

## Abstract

**Background:**

Anti-programmed cell death receptor-1 (PD-1) monotherapy is the standard treatment for metastatic melanoma in current. Camrelizumab is a humanized IgG4 anti-PD-1 monoclonal antibody whose safety and efficacy have not been reported in advanced Asian melanoma patients.

**Methods:**

This phase I study investigated the safety, activity, and pharmacokinetics of camrelizumab in Chinese patients with advanced melanoma. The study included two phases, the dose-escalation phase (“3 + 3” design at 60 mg, 200 mg, and 400 mg) and the dose-expansion phase.

**Results:**

No dose-limiting toxicities were recorded over the dose-escalation phase, and the maximum tolerated dose was not reached. The most common treatment-related adverse events (TRAEs) in 36 patients were reactive cutaneous capillary endothelial proliferation, followed by rash, fever, hypothyroidism, hyperthyroidism, vitiligo, and fatigue. Five grade 3 or above TRAEs were reported (13.9%), including two cases of elevated γ-glutamyltransferase and blood triglycerides without clinical symptoms, and one liver injury recovered after symptomatic treatment. The confirmed overall response rate was 13.9% (95%CI: 4.7, 29.5%) and disease control rate was 38.9% (95%CI: 23.1, 56.5%). The median progression-free survival was 1.8 months (95%CI: 1.1, 2.4) and the median overall survival was 11.1 months (95%CI: 6.8, 15.4).

**Conclusions:**

Camrelizumab had acceptable tolerability and similar anti-tumor activity compared with other anti-PD-1 antibodies in advanced Asian melanoma patients.

**Trial registration:**

ClinicalTrials.gov identification: NCT02738489. Registered on 14/04/2016, prospectively registered.

**Supplementary Information:**

The online version contains supplementary material available at 10.1186/s12885-022-09663-5.

## Introduction

Immune checkpoint inhibitors have revolutionized the treatment of advanced melanoma. The representative drugs include monoclonal antibodies against programmed cell death receptor-1 (PD-1) [1, 2], cytotoxic T-lymphocyte-associated antigen 4 (CTLA-4) [3], and lymphocyte-activation gene 3 (LAG3) [4], which inhibit T cell activity and contribute to immune evasion from the tumor. Nivolumab and pembrolizumab are recommended for the treatment of metastatic cutaneous melanoma in the National Comprehensive Cancer Network (NCCN) guideline, monotherapy or combined with ipilimumab [5].

Camrelizumab is a humanized IgG4-kappa anti-PD-1 monoclonal antibody. It was first approved in China for Hodgkin’s lymphoma, and later extended to advanced hepatocellular carcinoma, non-small cell lung cancer, esophageal squamous cell carcinoma, and nasopharyngeal carcinoma successively [6–10]. However, there are currently no efficacy and safety data reported in melanoma about camrelizumab.

In April 2016, a phase I clinical trial on the safety and tolerability of camrelizumab in patients with advanced melanoma (NCT02738489) was initiated at Peking University Cancer Hospital. In the first in human study conducted in Australia in 2015, camrelizumab was administrated in patients with solid tumors at 1 mg/kg, 3 mg/kg, 6 mg/kg, and 10 mg/kg every 2 weeks, and showed acceptable safety profile and potential antitumor activity [11]. Considering the safety data reported and convenience of drug administration, a fix dose of 60 mg, 200 mg and 400 mg Q2W were selected for this trial. This study aimed to summarize the safety and activity data of camrelizumab in the treatment of advanced melanoma to provide a basis for clinical application and subsequent trial design.

### Patients and methods

This study is an open-label, single-arm, and single-center trial approved by the Ethics Committee of Peking University Cancer Hospital. Patients with unresectable or metastatic melanoma (included acral, mucosal, uveal, cutaneous or other melanoma) who had progressed after standard treatment (cytotoxic drugs and/or anti-angiogenic agent at the time of study), or without effective treatment were allowed to participate. Other enrollment criteria included an Eastern Cooperative Oncology Group Performance Score (ECOG PS) of 0-1, without prior anti-PD1 agents or other immune checkpoint inhibitors, adequate hematologic, hepatic, and renal function, and measurable lesions defined by Response Evaluation Criteria In Solid Tumors (RECIST), version 1.1. Detailed inclusion and exclusion criteria were presented in the Additional file 1. All patients have signed informed consent forms. The study was conducted in accordance with the Declaration of Helsinki.

### Study design

This trial was registered prospectively at ClinicalTrials.gov (ID, NCT02738489) on 14/04/2016. The study is divided into two phases, dose-escalation and subsequent dose-expansion phase. In the first stage, the traditional “3 + 3” mode was adopted, and three fixed doses of camrelizumab were given, including 60 mg, 200 mg, and 400 mg every 2 weeks, respectively. If one dose-limited toxicity (DLT) occurs in the first cycle of three subjects, then three more subjects will be added. If DLT is not observed in the last three subjects, we moved to the next dose group. If one or more DLT occurs again in the last three subjects, the dose escalation is terminated, and the previous dose is defined as the maximum tolerated dose (MTD). No escalation was conducted beyond 400 mg even if MTD was not reached.

The primary endpoint of the study was the safety and tolerability of camrelizumab in patients with advanced melanoma. The secondary endpoints included pharmacokinetics (PK) and pharmacodynamics (PD) parameters of camrelizumab, anti-drug antibody (ADA) produced by patients, and anti-tumor activity of camrelizumab for advanced melanoma, including objective response rate (ORR), disease control rate (DCR), progression-free survival (PFS), and overall survival (OS). PFS was defined as the time from camrelizumab initiation to disease progression determined by RECIST 1.1 or death, whichever came first. OS was defined as the time from camrelizumab initiation to death due to any cause.

DLT was defined in this study as follows: ≥ grade 3 hematologic toxicities, ≥ grade 4 non-hematologic toxicities, grade 3 non-hematologic toxicities which demanded medical intervention, hospitalization, or lasted more than 3 days with best supportive care, and grade 3 laboratory abnormities with clinical significance. DLT was observed after the first administration in 4 weeks. The administration of camrelizumab was repeated every 2 weeks afterward, and the whole DLT observation period was 56 days. After the observation period of a dose group without DLT, the second phase of expansion at this dose can be carried out simultaneously with the subsequent dose escalation. Treatment continued until intolerable toxicity, progressive disease, or withdrawal of consent.

### Safety evaluation

The severity of adverse reactions was graded according to the National Cancer Institute Common Terminology Criteria for Adverse Events (NCI CTCAE), version 4.03. Delayed DLT (occurring after the DLT observation period) would not influence the dose escalation, but the ethics committee will be notified for further consideration. Delayed DLT in this study mainly referred to autoimmune reactions, including immune-related pneumonia, uveitis, colitis, aseptic meningitis, pancreatitis, hepatitis, nephritis, hyperthyroidism, hypothyroidism, hypophysitis, and hyperglycemia.

### Study assessments

Imaging evaluation was performed every two cycles, with every two administrations as one cycle. Objective response rate was assessed based on the RECIST 1.1 criteria by investigator. For clinically stable patients who have progressed in the initial radiological evaluation, treatment beyond disease progression was allowed according to the investigator’s choice.

### Pharmacokinetics and pharmacodynamics

Blood samples were collected after the first administration at pre-dose (in 0.5 h), 5 min (±5 min), 2 h (±10 min), 6 h (±10 min), 24 h (±30 min), 48 h (±30 min), day 8 (±60 min), day 15 (±60 min), day 22 (±60 min), and pre-dose (in 0.5 h), 5 min (±5 min) on day 1 and day 15 after multiple administrations for analyses of PK and receptor occupancy (RO) rate. PK parameters included half-life time(t_1/2_), time to maximum concentration (T_max_), maximum concentration (C_max_), area under the concentration-time curve, and the accumulation ratio R_ac_ calculated by C_max_. RO rate was defined as the ratio of PD-1 receptor occupied by camrelizumab on peripheral CD3 + T lymphocytes by flow cytometry.

### Statistical analysis

SAS software version 9.4 was used for statistical analysis. Counting data is represented by the number of cases or percentages, and measurement data is described as mean ± standard deviation (SD) or median (range). The PK and PD parameters were tabulated and analyzed descriptively, and presented using median and coefficient of variation (CV). The PK parameters were calculated using non-compartmental approaches by WinNonlin 7.0 software. The PD parameters were calculated using SAS software. The Clopper-Pearson method was used to estimate the two-sided 95% confidence interval (CI) for ORR and DCR. The survival curve was generated and analyzed by the Kaplan-Meier method.

## Results

From April 2016 to October 2017, a total of 36 patients with advanced melanoma were enrolled in this study. In the first stage, nine patients (three in each dose group) were included in the DLT analysis set. In the second stage, nine patients in each dose group were expanded. Twelve patients were eventually enrolled in each dose group and received at least one dose, and 33 patients received at least one efficacy evaluation.

There were 17 male (47.2%) and 19 female (52.8%) patients, with a median age of 52 (range: 29-68) years old. All patients had advanced melanoma who had failed previous first-line treatment. Primary sites included melanoma from acral (50.0%), mucosal (27.8%), cutaneous and unknown origins (19.4%), and 1 case from choroid.

Half of the patients were staged as M1c and M1d (brain metastasis stable for more than 2 months) by the American Joint Committee on Cancer (AJCC) 8th edition. The most common metastatic sites involved lymph nodes (77.8%), lung (58.3%), liver (33.3%), bone (19.4%), subcutaneous tissue (16.7%), and others. *BRAF* V600E mutation was detected in three patients (8.3%). More than half (66.7%) of the patients had previously received at least two lines of treatment of chemotherapy or targeted therapy. Most of the patients (77.8%) had an ECOG PS of 1 and elevated lactate dehydrogenase (LDH) levels (55.6%). Baseline characteristics are demonstrated in Table 1.

**Table 1:** Patient Characteristics

Characteristics	60 mg (*n =* 12)	200 mg (*n =* 12)	400 mg (*n =* 12)	Total (*n =* 36)
Age, years, median (range)	48 (31-68)	47.5 (29-67)	58.5 (47-65)	52 (29-68)
Sex, n (%)				
Male	7 (58.3)	5 (41.7)	5 (41.7)	17 (47.2)
Female	5 (41.7)	7 (58.3)	7 (58.3)	19 (52.8)
Primary site, n (%)				
Acral	6 (50.0)	6 (50.0)	6 (50.0)	18 (50.0)
Mucosal	1 (8.3)	4 (33.3)	5 (41.7)	10 (27.8)
Cutaneous	1 (8.3)	1 (8.3)	1 (8.3)	3 (8.3)
Unknown	3 (25.0)	1 (8.3)	0	4 (11.1)
Uveal	1 (8.3)	0	0	1 (2.8)
Stage^a^, n (%)				
M1a	4 (33.3)	5 (41.7)	3 (25.0)	12 (33.3)
M1b	2 (16.7)	1 (8.3)	3 (25.0)	6 (16.7)
M1c	5 (41.7)	5 (41.7)	5 (41.7)	15 (41.7)
M1d	1 (8.3)	1 (8.3)	1 (8.3)	3 (8.3)
Metastatic sites, n (%)				
Lymph nodes	8 (66.7)	11 (91.7)	9 (75.0)	28 (77.8)
Lung	8 (66.7)	5 (41.7)	8 (66.7)	21 (58.3)
Liver	4 (33.3)	4 (33.3)	4 (33.3)	12 (33.3)
Bone	4 (33.3)	1 (8.3)	2 (16.7)	7 (19.4)
Subcutaneous tissue	2 (16.7)	3 (25.0)	1 (8.3)	6 (16.7)
Others	8 (66.7)	6 (50.0)	2 (16.7)	16 (44.4)
*BRAF* status, n (%)				
Mutated	1 (8.3)	1 (8.3)	1 (8.3)	3 (8.3)
Wildtype	11 (91.7)	11 (91.7)	11 (91.7)	33 (91.7)
ECOG PS, n (%)				
0	0	4 (33.3)	4 (33.3)	8 (22.2)
1	12 (100.0)	8 (66.7)	8 (66.7)	28 (77.8)
Prior therapy lines, n (%)				
1	2 (16.7)	4 (33.3)	6 (50.0)	12 (33.3)
≥ 2	10 (83.3)	8 (66.7)	6 (50.0)	24 (66.7)
LDH, n (%)				
Normal	3 (25.0)	5 (41.7)	8 (66.7)	16 (44.4)
Elevated	9 (75.0)	7 (58.3)	4 (33.3)	20 (55.6)

*ECOG PS* Eastern Cooperative Oncology Group Performance Score, *LDH* Lactate dehydrogenase

^a^ Evaluated by the American Joint Committee on Cancer 8th edition

### Safety analysis

All 36 patients received at least one dose of camrelizumab. Thirty-three patients completed ≥2 dosing cycles, seven patients kept on treatment for more than 1 year, and four continued over 2 years. No DLT was observed in the three dose groups, and MTD was not reached. There were 29 cases (80.6%) that had at least one treatment related adverse event (TRAE), including 63.9% reactive cutaneous capillary endothelial proliferation (RCCEP), 13.9% rash, 11.1% fever, 25.0% hypothyroidism, 8.3% hyperthyroidism, and 5.6% vitiligo and fatigue, respectively. Adverse events of grade 3 or above included 1 case of liver injury, which recovered after symptomatic treatment, and 2 cases of elevated γ-glutamyltransferase and blood triglycerides without clinical symptoms. No administrations were suspended or terminated due to TRAEs. There were no study drug-related deaths (Table 2).

**Table 2:** Most common treatment related adverse events with grade 1 or above

Events, n (%)	60 mg (*n =* 12)	200 mg (*n =* 12)	400 mg (*n =* 12)	Total (*n =* 36)
All TRAE	7 (58.3)	10 (83.3)	12 (100.0)	29 (80.6)
TRAEs observed in ≥5% of patients				
RCCEP	3 (25)	8 (66.7)	12 (100)	23 (63.9)
Hypothyroidism	4 (33.3)	4 (33.3)	1 (8.3)	9 (25.0)
Abnormal hepatic function	1 (8.3)	1 (8.3)	4 (33.3)	6 (16.7)
Rash	2 (16.7)	2 (16.7)	1 (8.3)	5 (13.9)
Hypertriglyceridemia	2 (16.7)	1 (8.3)	2 (16.7)	5 (13.9)
Pyrexia	1 (8.3)	2 (16.7)	1 (8.3)	4 (11.1)
Hyperthyroidism	0	2 (16.7)	1 (8.3)	3 (8.3)
Vitiligo	0	2 (16.7)	0	2 (5.6)
Fatigue	0	1 (8.3)	1 (8.3)	2 (5.6)
Increased γ-glutamyltransferase	2 (16.7)	0	1 (8.3)	3 (8.3)

*RCCEP* Reactive cutaneous capillary endothelial proliferation, *TRAE* Treatment related adverse event

About RCCEP, most occurred after the first cycle of camrelizumab (2-4 weeks), predominantly on the face and trunk (with one case involving oral mucosa), and disappeared spontaneously after camrelizumab discontinuation (1-2 months). The incidence of RCCEP increased with the dose escalation of camrelizumab. All of RCCEP toxicities were grade 1 or 2, and camrelizumab was recommended to be continued.

### Anti-tumor activity

**Fig. 1:**
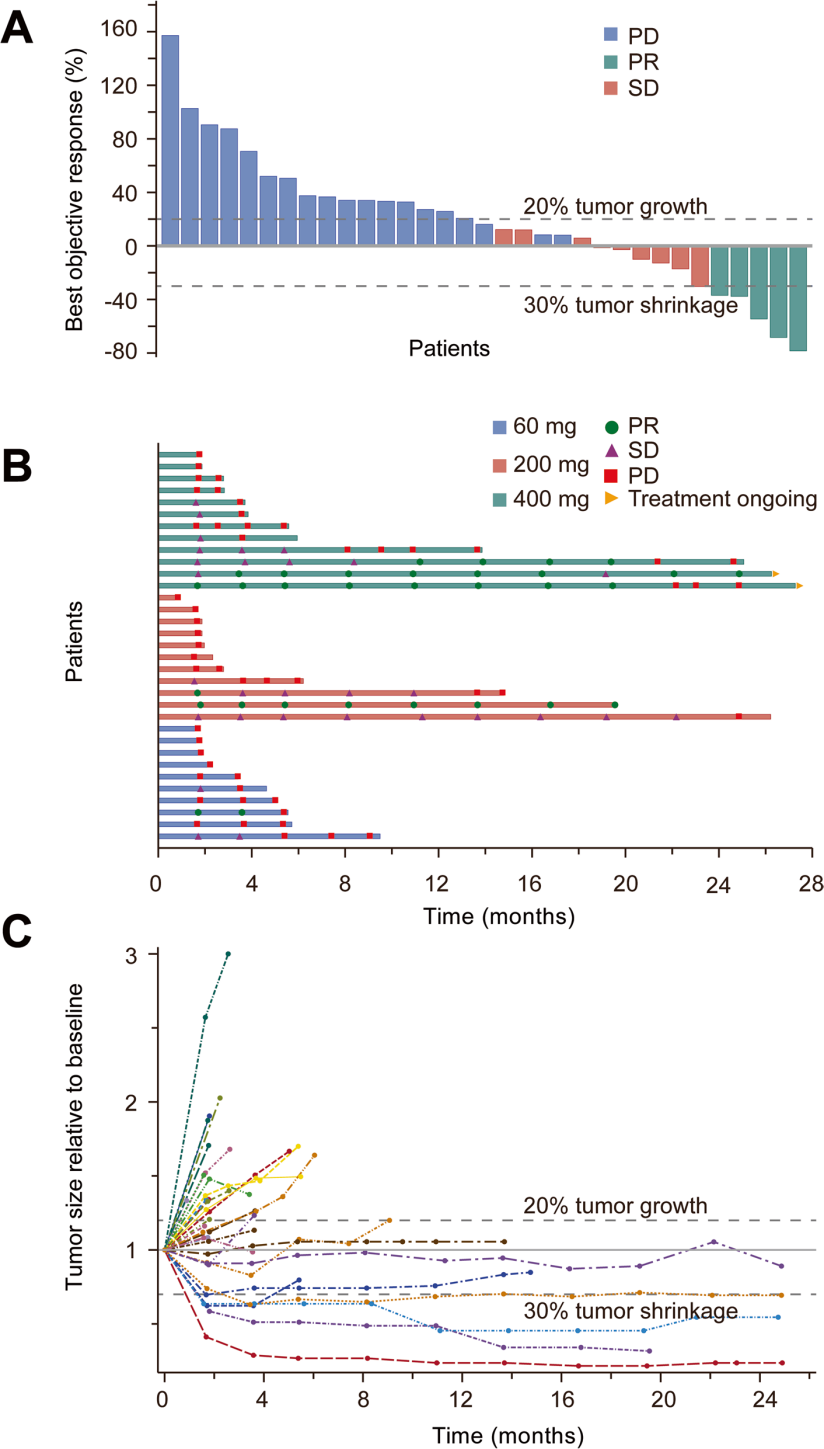
Anti-tumor activity of camrelizumab in patients with advanced melanoma. **A**. The best change from baseline in the sum of the longest target lesion diameters per Response Evaluation Criteria in Solid Tumors (RECIST) version 1.1. **B**. Exposure and duration of disease control. **C**. Change in the sum of the longest target lesion diameters over time from baseline

**Fig. 2:**
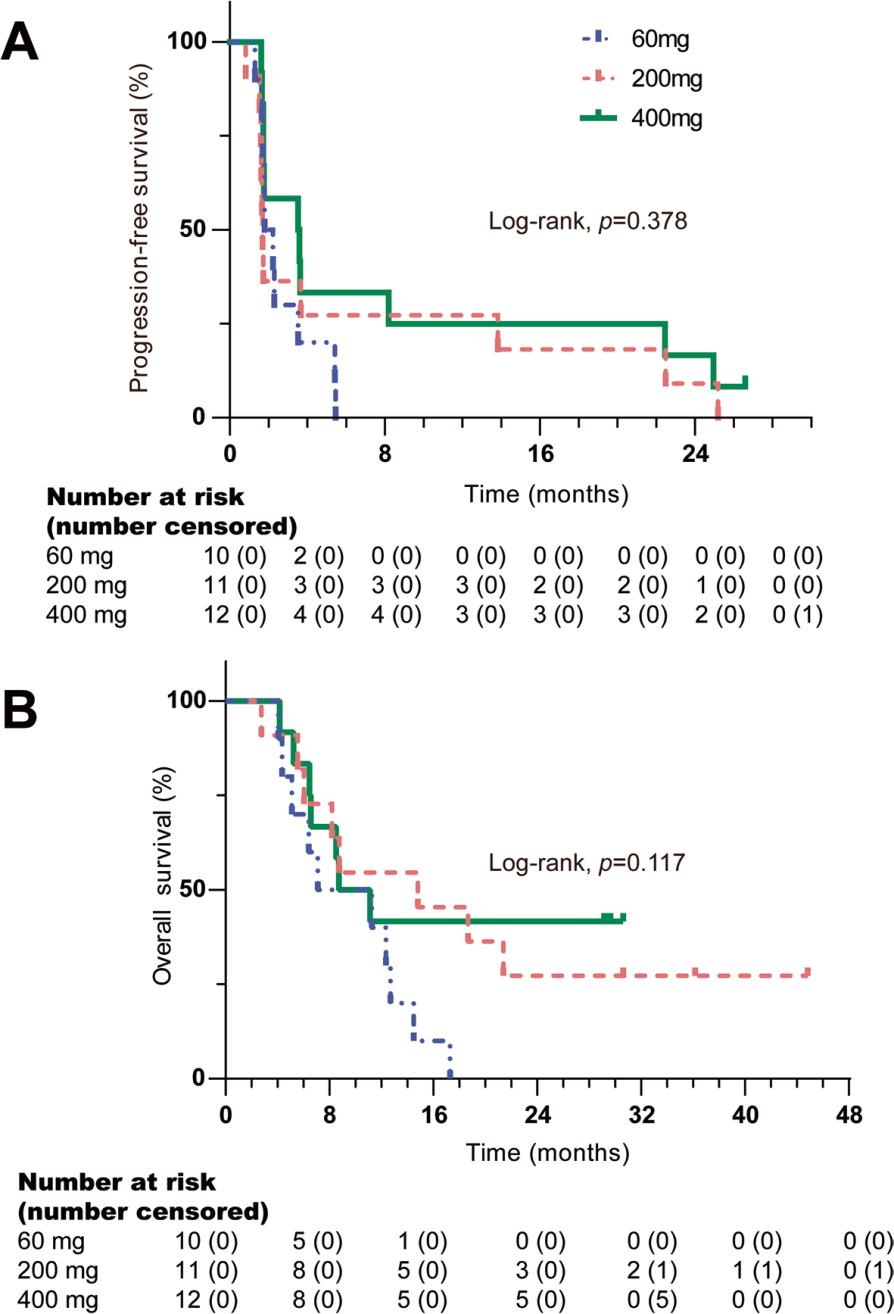
The Kaplan-Meier curves of patients received a dose of 60, 200 or 400 mg of camrelizumab. **A** Progression-free survival; **B** Overall survival (two-sided log-rank test)

As of April 1, 2020, the median follow-up time was 30.6 months (95%CI: 29.8-31.4). Among the 36 patients, three were not evaluated. Five patients achieved confirmed PR, including one case from an unknown primary origin in the 60 mg dose group, one mucosal melanoma in the 200 mg dose group, and two acral and one mucosal melanoma in the 400 mg dose group. There were 9 cases of SD and 19 PD, as was shown in Fig. 1. Based on the full analysis dataset, ORR was 13.9% (95%CI: 4.7, 29.5%) and DCR was 38.9% (95%CI: 23.1, 56.5%). The median PFS was 1.8 months (95%CI: 1.1, 2.4) and the median OS was 11.1 months (95%CI: 6.8, 15.4) (Table 3, Fig. 2). For 33 patients who had efficacy evaluation, the confirmed ORR was 15.2%.

**Table 3:** Anti-tumor activity of camrelizumab in different dosage groups

Variables	60 mg (*n =* 12)	200 mg (*n =* 12)	400 mg (*n =* 12)	Total (*n =* 36)
Best response, n (%)				
PR	1 (8.3)	1 (8.3)	3 (25.0)	5 (13.9)
SD	2 (16.7)	3 (25.0)	4 (33.3)	9 (25.0)
PD	7 (58.3)	7 (58.3)	5 (41.7)	19 (52.8)
Missing	2 (16.7)	1 (8.3)	0	3 (8.3)
ORR, n (%)	1 (8.3)	1 (8.3)	3 (25.0)	5 (13.9)
95% CI	0.2-38.5	0.2-38.5	5.5-57.2	4.7-29.5
DCR, n (%)	3 (25.0)	4 (33.3)	7 (58.3)	14 (38.9)
95% CI	5.5-57.2	9.9-65.1	27.7-84.8	23.1-56.5
PFS, months, median (95% CI)	1.8 (1.2-2.3)	1.7 (1.6-1.8)	3.5 (0.4-6.6)	1.8 (1.1-2.4)
OS, months, median (95% CI)	7.1 (0-14.6)	14.8 (3.5-26.1)	8.7 (4.3-13.1)	11.1 (6.8-15.4)

*PR* Partial response, *SD* Stable disease, *PD* Progressive disease, *ORR* Objective response rate, *DCR* Disease control rate, *PFS* Progression-free survival, *OS* Overall survival, *CI* Confidence interval

### Pharmacokinetics and pharmacodynamics

The average concentration of camrelizumab in melanoma patients increased from a dose of 60 mg to 400 mg. The peak blood concentration was reached at about 0.7 h after the first administration of camrelizumab in each dose group and then gradually decreased. The serum exposure level of camrelizumab increased with dose escalation. The average values ​​of C_max_ were 19.93, 73.93, and 139.69 μg/mL from 60 mg to 400 mg; the average values ​​of AUC_0-t_ were 68.77, 400.69, and 1044.37 day·μg/mL, and the average values ​​of AUC_0-inf_ were 68.46, 452.31, and 1285.45 day·μg/mL, respectively. After multiple administrations, the accumulation ratio Rac calculated by C_max_ was 1.11, 1.50, and 1.37, respectively, indicating that the peak serum concentration of camrelizumab increased slightly after multiple administrations. However, there was no significant accumulation in each dose group. In the dose range from 60 mg to 400 mg of camrelizumab, the increase of C_max_ was in a linear relationship with the increase of dose, while the increase ratio of AUC was greater than that of dose (Table 4).

**Table 4:** Pharmacokinetic parameters of camrelizumab following a single dose

Parameters (CV%)	60 mg (*n =* 12)	200 mg (*n =* 12)	400 mg (*n =* 12)
C_max_ (ug/ml) mean ± SD, (%CV)	19.93 ± 4.21 (21.1)	73.93 ± 11.09 (15.0)	139.69 ± 23.49 (16.8)
T_max_ (hours), median (range)	0.73 (0.63, 48.42)	0.71 (0.65, 24.65)	0.78 (0.65, 6.72)
AUC_0-t_ (day·ug/ml), median (%CV)	68.77 (38.8)	400.69 (22.5)	1044.37 (18.9)
AUC_0-inf_ (day·ug/ml), median (%CV)	68.46 (39.2)	452.31 (27.8)	1285.45 (23.6)
t_1/2_, day, median (%CV)	3.06 (31.1)	5.92 (27.8)	12.22 (25.8)
R_ac_,c_max_, median (%CV)	1.11 (21.4)	1.50 (7.3)	1.37 (10.3)

*SD* Standard deviation, *CV* Coefficient of variation, *C*_*max*_ Maximal plasma concentration, *T*_*max*_ Time to maximal plasma concentration, *AUC*_*0-t*_ Area under the concentration-time curve from 0 to the last measurable concentration, *AUC*_*0-inf*_ Area under the concentration-time curve from 0 to infinite time; t½ terminal half-life, *Rac*,*c*_*max*_ The accumulation ratio calculated by *C*_*max*_

The receptor occupancy rate of camrelizumab in different dose groups was about 66% ~ 71% after a single dose of camrelizumab, which indicated that PD-1 receptors were quickly occupied by camrelizumab. The average PD-1 receptor occupancy rate is maintained at more than 55% from 24 h to day 29 after administration. After multiple administrations of 3 ~ 5 cycles of camrelizumab, the receptor occupancy rate nearly stabilized. The occupancy rate of the receptors remained above 55% in each dose group when the patients discontinued treatment.

## Discussion

In recent years, immunotherapy has made a breakthrough in the treatment of melanoma. PD-1 and PD-L1 monoclonal antibodies have changed the treatment pattern of melanoma. Camrelizumab is a humanized IgG4 anti-PD-1 monoclonal antibody whose efficacy and safety have been reported in multiple tumor types [6–10]. This study is the first phase I clinical study of camrelizumab reported on melanoma.

In this study, camrelizumab is safe and well-tolerated in patients with advanced melanoma. DLT was not observed, 29 patients (80.6%) had at least one TRAE, and most of the TRAEs were mild to moderate. No administrations were suspended or terminated due to TRAEs and no TRAEs led to death. The common adverse reactions reported by nivolumab and pembrolizumab included fatigue, rash, pruritus, diarrhea, nausea, hypothyroidism, hyperthyroidism, loss of appetite, pneumonia, abdominal pain, constipation, musculoskeletal pain, weight loss, infusion-related reactions, and so on [2, 12]. In addition to RCCEP, the types of common TRAEs of camrelizumab are similar to those reported by nivolumab and pembrolizumab. The common manifestations of camrelizumab-induced toxicities are hypothyroidism and skin reactions, including RCCEP, rash, and vitiligo. Adverse events of grade 3 or higher included increased γ-glutamyltransferase and increased blood triglycerides without clinical symptoms in 2 cases and 1 case of liver injury, recovered without using glucocorticoid. Immune-related adverse events such as pneumonia, colitis, or hypophysitis were not observed, possibly due to the small number of cases.

The skin toxicity of camrelizumab is different from other anti-PD-1 antibodies, distinguished by RCCEP, which is probably due to the different binding sites. Camrelizumab induced RCCEP is an adverse skin reaction related to excessive immune activation, with infiltration of CD4+ T cells and M2 macrophages and the release of cytokines in local skin tissues [13]. In this study, 23 patients (63.9%) developed RCCEP, and all were grade 1-2 and clinically manageable. In previous studies, the majority of RCCEP caused by camrelizumab are grade 1 or 2, and RCCEP with grade 4 or higher has not been reported [13]. For RCCEP with grade 1 or 2, it was recommended to continue camrelizumab, and for grade 3 RCCEP, suspend the camrelizumab until RCCEP has recovered to grade 1. Interestingly, previous studies showed that the occurrence of RCCEP induced by camrelizumab is associated with the better prognosis (including objective response and survival benefit) [9, 14, 15]. The molecular mechanism by which camrelizumab induces Th2 cells and M2 macrophages differentiation in the dermis and promotes blood vessel growth is being explored.

The PD-1 receptor occupancy rate refers to the percentage of PD-1 receptors occupied by camrelizumab on the surface of peripheral T lymphocytes. Camrelizumab inhibits the binding of T cells to the ligand PD-L1 on the surface of tumor cells by binding to the PD-1 receptor on the surface of T cells, relieves tumor cells from inhibiting the body’s immune function, and activates immune response to kill tumor cells. Under the frequency of multiple doses at Q2W, the receptor occupancy rate of each dose group is relatively stable, and most of the ratios ​​remained between 50 to 75%. The receptor occupancy rate has no obvious correlation among different doses. As PD-1 receptor occupancy is the theoretical premise of anti-tumor activity, this result suggests that camrelizumab can fully occupy the PD-1 receptor and block the PD-1/PD-L1 signal under Q2W administration frequency. Besides, the expansion cohort of first in human trial conducted in Australia demonstrated that camrelizumab showed manageable toxicity at a fix dose of 200 mg [11]. Several studies in China have shown that camrelizumab 200 mg showed acceptable safety profile and potential efficacy in various tumors [6, 10, 16–19]. Taken together, the fixed dose of 200 mg could be the recommended dose in the further trials.

At present, only pembrolizumab and toripalimab have been approved for advanced melanoma patients after failure to first-line treatment in China. Asian melanomas are mainly derived from acral and mucosal origins, which were less responsive to immunotherapy than cutaneous or unknown primary subtypes [20, 21]. Even in cutaneous subtypes, the efficacy of anti-PD-1 monoclonal antibody in East-Asians is significantly lower than Caucasians according to a recent retrospective study [22]. The Keynote-151 study included 103 patients with advanced melanoma in China treated with pembrolizumab after chemotherapy [23]. The ORR was 16.7%, DCR was 38.2%, median PFS was 2.8 months, and the median OS was 12.1 months. The POLARIS-01 study enrolled 128 Chinese melanoma patients treated with toripalimab after first-line treatment failure [24]. The ORR was 17.3%, DCR was 57.5%, median PFS was 3.6 months, and median OS was not yet reported. This phase 1 study of camrelizumab enrolled 36 patients with advanced melanoma, mainly acral and mucosal melanoma (77.8%). Regardless of different dose groups of camrelizumab, the ORR of evaluated patients was confirmed to reach 15.2%. The median PFS was 1.8 months, and the median OS was 11.1 months. The efficacy and survival data were similar to other anti-PD-1 antibodies in advanced Asian melanoma patients.

Acral and mucosal subtypes are more aggressive than cutaneous melanoma and less responsive to anti-PD-1 monotherapy. According to previous sequencing studies, genetic aberrations in CDK and PI3K/AKT/mTOR pathways are frequently detected in acral and mucosal melanoma subtypes [25–27]. Common structural and copy number variations instead of single nucleotide variations in these patients may contribute to the relatively low response rates of immunotherapy [28, 29]. Current studies are mainly focused on combination therapy. In acral melanoma, camrelizumab combined with apatinib, an anti-angiogenesis agent, had an increased ORR of 22% in treatment-naïve patients [30]. More exploratory studies are demanded to improve the outcome of Asian melanoma patients.

In summary, camrelizumab had acceptable tolerability and approximate efficacy compared with other anti-PD-1 antibodies in advanced Asian melanoma patients. Camrelizumab at a fixed dose of 200 mg Q2W was recommended for clinical application and further clinical trials.

## Supplementary Information

**Additional file 1.**

## Data Availability

The datasets analyzed during the current study are not publicly available due to hospital policy but are available on reasonable request by contacting Dr. Li Zhou (zhoulilucky@126.com).

## Abbreviations

PD-1: Programmed cell death receptor-1
CTLA-4: Cytotoxic T-lymphocyte-associated antigen 4
LAG3: Lymphocyte-activation gene 3
RECIST: Response Evaluation Criteria In Solid Tumors
NCI CTCAE: National Cancer Institute Common Terminology Criteria for Adverse Events
DLT: Dose-limited toxicity
MTD: Maximum tolerated dose
PK: Pharmacokinetics
PD: Pharmacodynamics
ECOG PS: Eastern Cooperative Oncology Group Performance Score
LDH: Lactate dehydrogenase
AJCC: American Joint Committee on Cancer
PR: Partial response
SD: Stable disease
PD: Progressive disease
ORR: Objective response rate
DCR: Disease control rate
CI: Confidence interval
PFS: Progression-free survival
OS: Overall survival
SD: Standard deviation
CV: Coefficient of variation
C_max_: Maximal plasma concentration
T_max_: Time to maximal plasma concentration
AUC_0-t_: Area under the concentration-time curve from 0 to the last measurable concentration
AUC_0-inf_: Area under the concentration-time curve from 0 to infinite time
t½: Terminal half-life
R_ac_,c_max_: The accumulation ratio calculated by C_max_
TRAE: Treatment related adverse event
RCCEP: Reactive cutaneous capillary endothelial proliferation
