# Supplementary material for: Safety, activity, and pharmacokinetics of camrelizumab in advanced Asian melanoma patients: a phase I study
Source: BMC Cancer. 2022 May 20;22:565. doi: 10.1186/s12885-022-09663-5 (PMC9123656; doi:10.1186/s12885-022-09663-5)
Supplement: Supplementary file 1 — Additional file 1. [file 12885_2022_9663_MOESM1_ESM.docx]

**Inclusion Criteria:**

1. Male or female patients aged 18–70 years old, who agree to provide pathological tumor biopsy specimens during the screening period and after the end of treatment;

2. Patients with pathologically confirmed advanced melanoma who have failed standard treatments or without effective treatment methods (e.g., chemotherapy, targeted therapy and immunotherapy other than those targeting PD-1/PD-L1);

3. ECOG PS score: 0-1;

4. Life expectancy ≥ 12 weeks;

5. With measurable and evaluable lesions complying with RECIST v1.1;

6. Major organ functions must meet the following rules (not including the use of any blood cell growth factors within 14 days before screening):

• Absolute neutrophil count (ANC) ≥ 1.5 × 10^9^/L;

• Platelets ≥ 100 × 10^9^/L;

• Hemoglobin ≥ 9 g/dL;

• Serum albumin ≥ 2.8 g/dL;

• Bilirubin ≤ 1.5 × ULN, ALT and AST ≤ 1.5 × ULN; if there is liver metastasis, ALT and AST ≤ 5 × ULN;

• Creatinine clearance ≥ 50 mL/min (calculated by standard Cockcroft-Gault formula);

7. For female patients of childbearing potential or female patients who are not sterilized by surgical operations, they need to use a medically approved contraceptive measure (such as an intrauterine device [IUD], contraceptives or condom) during the study treatment period and within 3 months after the end of the study treatment; for female patients of childbearing potential who are not sterilized by surgical operations, they must have a negative serum or urine HCG test result within 7 days prior to study enrollment; and they must not be in the lactation period;

8. Signed the informed consent form.

**Exclusion Criteria:**

1. Patients with any active autoimmune diseases or a history of autoimmune diseases (including but not limited to the following: interstitial pneumonia, uveitis, enteritis, hepatitis, hypophysitis, nephritis, vasculitis, hyperthyroidism, hypothyroidism; adult patients with vitiligo or completely relieved childhood asthma can be enrolled if they do not require any intervention; patients with asthma requiring medical intervention with bronchodilators cannot be enrolled);

2. Patients who are currently using immunosuppressive agents, or systemic or absorbable local hormonal therapies for immunosuppression purposes (> 10 mg/day prednisone or equivalent) and still use the above drugs within 2 weeks prior to enrollment;

3. Patients who are known to be previously allergic to macromolecular protein preparations or any component of camrelizumab;

4. Patients with clinically symptomatic metastases to central nervous system (e.g., cerebral edema requiring hormonal intervention, or progression of brain metastasis). Patients who have received treatments for brain or meningeal metastasis can be included if they are clinically stable (MRI) for at least 2 months and have discontinued systemic hormonal therapy (> 10 mg/day prednisone or equivalent) for more than 2 weeks;

5. Patients with clinical symptoms or diseases of the heart that are not well controlled, such as (1) > NYHA Grade II cardiac failure, (2) unstable angina, (3) myocardial infarct within the past year, and (4) clinically significant supraventricular or ventricular arrhythmia requiring treatment or interventions;

6. Patients who have previously received radiotherapy, chemotherapy, hormone therapy, surgery or molecular targeted therapy with an interval of less than 4 weeks from the completion of the treatment to the study medication (for patients who have previously received chemotherapy with nitrosourea or mitomycin, the interval from the end of chemotherapy to the study enrollment is less than 6 weeks); patients whose adverse events caused by previous treatments have not recovered to CTCAE Grade ≤ 1;

7. Patients with active infection or unexplained fever > 38.5 °C during screening or prior to the first dose (patients with tumor-induced fever may be enrolled as per the judgment of the investigator);

8. Patients with congenital or acquired immunodeficiency (such as HIV, HBV, and HCV infections);

9. Patients who are participating in other clinical studies or have just completed a previous clinical study within less than a month;

10. Patients who may receive other systemic anti-tumor treatments during the study;

11. Patients who have previously received other PD-1 antibody treatments or other immunotherapies targeting PD-1/PD-L1;

12. Patients with a known history of psychotropic substance abuse, alcohol abuse, or drug abuse;

13. The investigator judges that the patient may have other factors leading to the termination of the study, such as other serious diseases (including mental illness) requiring concomitant treatment, serious laboratory test abnormalities, accompanied by family or social factors that can affect the safety of the patient or the collection of study data and samples.
